# Study of Query Expansion Techniques and Their Application in the Biomedical Information Retrieval

**DOI:** 10.1155/2014/132158

**Published:** 2014-03-02

**Authors:** A. R. Rivas, E. L. Iglesias, L. Borrajo

**Affiliations:** Department of Computer Science, Higher Technical School of Computer Engineering, University of Vigo, 32004 Ourense, Spain

## Abstract

Information Retrieval focuses on finding documents whose content matches with a user query from a large document collection. As formulating well-designed queries is difficult for most users, it is necessary to use query expansion to retrieve relevant information. Query expansion techniques are widely applied for improving the efficiency of the textual information retrieval systems. These techniques help to overcome vocabulary mismatch issues by expanding the original query with additional relevant terms and reweighting the terms in the expanded query. In this paper, different text preprocessing and query expansion approaches are combined to improve the documents initially retrieved by a query in a scientific documental database. A corpus belonging to MEDLINE, called Cystic Fibrosis, is used as a knowledge source. Experimental results show that the proposed combinations of techniques greatly enhance the efficiency obtained by traditional queries.

## 1. Introduction

Biomedical knowledge is growing at a high pace, and large collections of publications offer an excellent opportunity for discovering hidden biomedical knowledge by applying information retrieval (IR) and related technologies. IR is related to the representation, storage, organization of, and access to the information items [1]. Information items must be represented in order to allow users to have easy access to the information of interest, and the user requirements must be presented in a good format to be translated into a query which can be processed by the search engine (or IR system). The translation is presented like a set of keywords (or index terms) which represents the query and summarizes the information in which the user is interested.

Information Retrieval using only keywords is not usually very efficient. In general, information about a particular issue can be represented with different keywords which may not coincide exactly with the terms entered in the query by the user. The user query can include keywords that are not present in documents, but documents could be relevant because they have other words with the same meaning. Using *query expansion* (QE) techniques, a query is reformulated to improve retrieval performance and obtain additional relevant documents by expanding the original query with additional relevant terms and reweighting the terms in the expanded query. Query expansion techniques are widely used for improving the efficiency of textual information retrieval systems, helping to overcome vocabulary mismatch issues including words in queries with the same or related meaning.

This paper analyzes different techniques of document preprocessing and query expansion in order to know which ones offer better results when applied to query biomedical databases. This research aims to improve the efficiency of the queries based on classic models (where documents are retrieved even if only a small part of them is related to the query), when they are performed in public scientific databases, such as Pubmed.

The remainder of the paper is organized as follows: Section 2 presents an overview of the general Information Retrieval process.  Section 3 describes the preprocessing and Query Expansion methods employed in this research for retrieving relevant documents and the experimental results. Finally, the conclusions are included in Section 4.

## 2. Information Retrieval Process

**Figure 1:**
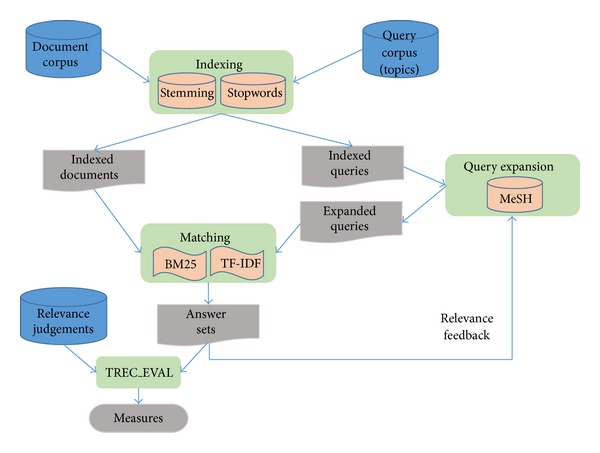
The information retrieval process.

In Information Retrieval, the query process is composed of two main phases, indexing and matching (see Figure 1). Additionally, it is possible to expand the queries to improve the efficiency of the retrieval.

The *indexing* step preprocesses documents and queries in order to obtain keywords (relevant words, also named *terms*) to be used in the query. At this point, it is important to consider the use of stemming and stopword lists in order to reduce related words to their stem, base or root form. This is achieved by launching affix removal to adapt different derivational or inflectional variants of the same word to a single indexing form and remove words that do not contain information relevant to the document [1, 2].

*Matching* is the process of computing the similarity between documents and queries by weighting terms, the most frequently applied algorithms being the TF-IDF and BM25 algorithms. Most retrieval systems return a ranked document list in response to a query, where the documents more similar to the query considered by the system are first on the list.

Once the first answer set is obtained, different *query expansion* techniques can be applied. For example, the most relevant keywords of the top documents previously retrieved can be added to the query in order to rerank the documents. This process is known as *relevance feedback*. The retrieval can be further enhanced by modifying the words of the queries using other keywords more representative of the document content (e.g., including MeSH Headings).

This study uses the Lemur language modeling toolkit (Lemur Project http://www.lemurproject.org/) for the indexing, mapping, and feedback processes. Lemur is a software tool designed to facilitate research in language modeling and IR, using weighting algorithms to provide methods for parsing queries, indexing documents, and retrieving documents related to queries.

In order to evaluate the results of the retrieval process, a program inside the TREC conference, trec_eval (trec_eval http://trec.nist.gov), is used. Trec_eval makes it possible to obtain measures such as the *Total number of documents over all queries* (Retrieved, Relevant and Rel_ret (relevant, and retrieved)) or *MAP*, *R-prec*, and *Interpolated Recall-Precision Averages*.

The following subsections introduce more details of the concepts related to document corpora, stemming, stopwords, weighting algorithms, query expansion, and measures.

### 2.1. Document Corpora

As seen in Figure 1, three document corpora are needed to analyze the efficiency of a query system: the original document corpus, the textual descriptions of the users queries (topics), and the relevant judgments given by the experts [3]. A document corpus represents a sampling of articles published. The format of the data uses a labeled bracketing, the topics are a description in natural language of the information that the user needs, typically one sentence, and, finally, the relevance judgments are done by potential users, called experts or judges.

Most of the public biomedical document corpora belong to MEDLINE, which is used in our study: *Cystic Fibrosis *(Cystic Fibrosis Collection http://grupoweb.upf.es/WRG/mir2ed/ref.php) (CF). It consists of 1239 documents published between 1974 and 1979 discussing various aspects of Cystic Fibrosis. Cystic Fibrosis documents are composed of the Abstract (AB), Title (TI), and a set of manually assigned MeSH (MeSH Home page http://www.nlm.nih.gov/mesh/) (Medical Subject Headings) of a MeSH thesaurus.

MeSH thesaurus is a controlled vocabulary used for indexing, cataloging, and searching for biomedical and health-related information and documents. It imposes uniformity to the indexing of the scientific literature. MeSH thesaurus contains approximately 26 thousand terms and is updated annually to reflect changes in medicine and medical terminology.

MeSH has a hierarchical structure with sets of terms, naming, *descriptors*, that allow searching at various levels of specificity. Expert annotators assign MeSH Headings terms to the documents in order to allow the user to retrieve the information that explains the same concept with different terminology. On average, 5 to 15 subject headings are assigned by document, of which 3 to 4 of them are major subjects (MJ) and the others are minor subjects (MN). Major MeSH terms describe the main topics of the document, and minor MeSH terms provide more details about it [4–8].

In Table 1, an example of MEDLINE document is showed. It contains the title (TI), the major subjects (MJ), the minor subjects (MN), and the document abstract (AB).

Each MeSH Heading is related to several Entry terms. *Entry terms* are synonyms, alternate forms, and other closely related terms with a given MeSH record. They are generally used interchangeably with the MeSH Heading for the purpose of indexing and retrieval, thus increasing the access points to MeSH indexed data.

The Cystic Fibrosis collection also contains 100 queries and the documents relevant to each query [1] (see Table 2). Further, four scores are provided for each relevant document. Relevance scores can be 0 (which indicates nonrelevance), 1 (which indicates marginal relevance), and 2 (which indicates high relevance).

### 2.2. Stemming

Stemming is the process of reducing related words to their stem, base or root form through affix removal. Its aim is to adapt different derivational or inflectional variants of the same word to a single indexing form [1, 2].

There are two major stemmers in use for English IR: the Porter stemmer and the Krovetz stemmer. *Porter Stemmer* is a process for removing suffixes from words, such as gerunds and plurals, and replacing inflectional endings [9]. It is composed of rules, each of which deals with a specific suffix and has certain conditions to satisfy. The suffixes of words are checked against each rule in a sequential manner until it matches one; the conditions in the rule are then tested, which may result in a suffix removal or modification.

Alternatively, *Krovetz Stemmer* removes inflectional suffixes in three steps: the conversion of a plural to its single form, the conversion of past to present tense, and the removal of *-ing*. The process firstly removes the suffix and then, through a process of checking in a dictionary, returns the stem to a word [10].

### 2.3. Stopwords

In Information Retrieval, a document is indexed by the frequency of its words. Statistical analysis of this process shows that some words have low frequency, while others have high frequency [11]. For example, *and*, *of*, and *the* appear frequently in the documents without significant information. This set of words is referred to as stopwords. Elimination of stopwords can significantly reduce the size of the indexing structure, speed up the calculation and increase accuracy. Up to now, a lot of stopword lists have been developed for the English language, for example, the US National Library of Medicines official list of stopwords (NLM stopword list http://www.netautopsy.org/umlsstop.htm), and the stopword list built by Gerard Salton and Chris Buckley for the experimental SMART Information Retrieval system (SMART stopword list http://www.lextek.com/manuals/onix/stopwords2.html).

### 2.4. Okapi BM25 Weighting Algorithm

Okapi BM25, or BM25, is a weighting function used to rank documents according to their relevance to a given query [12]. Many researchers apply the BM25 function in different corpus to retrieve relevant documents.

BM25 is a probabilistic model, where the weight of a search term is assigned based on its frequency within the document and the frequency of the query term. The corresponding weighting function is as follows:

(1) wi=SJ·(k1+1)·freqidk1·[(1−b)+b·(dl/avdl)]+freqid ·(k3+1)·freqiqk3+freqiq;

- *k*_1_, *b*, and *k*_3_ are parameters which depend on the queries and the database;
- freq_*id*_ is the occurrence frequency of the term in the document *d*;
- freq_*iq*_ is the frequency of the term in the topic from which the query *q* is derived;
- *dl* and *a* *v* *dl* are, respectively, the document length and the average document length in the corpus.

SJ is the Robertson Sparck Jones weight [13], calculated as

(2) $$\begin{array}{c} \log\frac{\left(r+0.5\right)/\left(R-r+0.5\right)}{\left(n-r+0.5\right)/\left(N-n-R+r+0.5\right)}, \end{array}$$

where *R* is the number of documents relevant to a specific topic, *r* is the number of relevant documents containing the term *i*, *N* is the total number of documents in the collection, and *n* is the number of documents containing the term.

### 2.5. TF-IDF Weighting Algorithm

The TF-IDF weighting algorithm (termed frequency-inverse document frequency) is often used in Information Retrieval and text mining [1]. This weight is a statistical measure used to evaluate the importance of a word to a document in a collection or corpus. The importance increases proportionally to the number of times a word appears in the document but is offset by the frequency of the word in the corpus.

Variations of the TF-IDF weighting scheme are often used by search engines as a central tool in scoring and ranking the document relevance given a user query [14, 15]. In our experiments, the tf formulas for the TF-IDF weighting algorithm applied are

 Raw TF formulas
  (3) $$\begin{array}{c} \text{t}{\text{f}}_{id}=\frac{\text{fre}{\text{q}}_{id}}{\text{ma}{\text{x}}_{l}\;\text{fre}{\text{q}}_{ld}},\\ \text{t}{\text{f}}_{iq}=\text{fre}{\text{q}}_{iq}, \end{array}$$

where max_*l*_ freq_*ld*_ represents the frequency of the most frequent term in the document *d*,

 Log TF formulas
  (4) tfid=log⁡⁡(Raw  TF+1),tfiq=log⁡⁡(Raw  TF+1),
 Okapi TF formulas
  (5) $$\begin{array}{c} \text{t}{\text{f}}_{id}=\frac{k_{1}\cdot \text{fre}{\text{q}}_{id}}{\text{fre}{\text{q}}_{id}+k_{1}\cdot \left(1-b+b\cdot \left(dl/avdl\right)\right)},\\ \text{t}{\text{f}}_{i\text{q}}=\frac{k_{1}\cdot \text{fre}{\text{q}}_{iq}}{\text{fre}{\text{q}}_{iq}+k_{1}\cdot \left(1-b+b\cdot \left(ql/avdl\right)\right)}, \end{array}$$

where *ql* is the query length.

Table 3 contains the correspondence between the parameters of the BM25 weighting algorithm and the TF-IDF with Okapi TF formulas, used in our experiments.

The IDF (inverse document frequency) function is as follows [1, 2]:

(6) $$\begin{array}{c} \text{idf}=\log\left(\frac{n}{N}+1\right). \end{array}$$

Thus, the weight of a term is calculated as

(7) $$\begin{array}{c} w_{i}=\text{t}{\text{f}}_{id}\cdot \text{t}{\text{f}}_{iq}\cdot \text{id}{\text{f}}_{i}^{2}. \end{array}$$

### 2.6. Query Expansion

Query expansion techniques have been studied for nearly three decades. The various methods proposed in the literature can be classified into the following three groups [16]: query specific, corpus specific, and language specific.

- *Query-specific terms* can be identified by locating new terms in a subset of the documents retrieved by a specific query. This is the approach taken by relevance feedback systems, where related terms come from the contents of user-identified relevant documents. This has been shown to be quite effective, but it requires that users indicate which documents are relevant. More recently, search improvements are being achieved [17, 18] without the user's relevance judgments.
- *Corpus-specific terms* are found by analyzing the contents of a particular full-text database to identify terms used in similar ways. It may be hand-built, a time-consuming and ad hoc process, or created automatically. Traditional automatic thesaurus construction techniques group words together based on their occurrence patterns at a document level [19, 20], that is, words which often occur together in documents are assumed to be similar. These thesauri can then be used for automatic or manual query expansion.
- *Language-specific terms* may be found from generally available online thesauri that are not tailored for any particular text collection. Liddy and Myaeng [21] use the Longman's Dictionary of Contemporary English, a semantically coded dictionary. Voorhees (1994) used WordNet [22], a manually constructed network of lexical relationships. Borrajo et al. [23] study the use of dictionaries in the classification of biomedical texts with three different dictionaries (BioCreative [24], NLPBA [25] and an ad hoc subset of the UniProt database named Protein [26]).

This research adopts an automatic query-specific terms approach for locating related terms. We are particularly interested in these techniques because they are commonly used to add useful words to a query. Unfortunately, casual users seldom provide a system with the relevance judgments needed in relevance feedback. In such situations, ad hoc or blind feedback is commonly used to expand the user query. This method takes the form of pseudorelevance feedback, where the actual input from the user is not required. In this method, a small set of documents is retrieved using the original user query; these documents are all assumed to be relevant without any intervention by the user [27]. The content of the assessed documents is used to adjust the weights of terms in the original query and/or to add keywords to the query. The new query is reformulated towards relevant documents and away from the nonrelevant ones [10, 28, 29].

The Lemur toolkit used in our experiments implements the Rocchio formulation for pseudo relevance feedback. It first applies the standard retrieval model [1, 2] to retrieve *m* documents *d*_1_,…, *d*_*m*_ for a given query *q*. Given the retrieved documents and the original query, the expanded query *q*′ is computed as

(8) $$\begin{array}{c} q^{\prime }=q+\frac{\alpha }{M}\sum_{i=1}^{M}d_{i}, \end{array}$$

where *M* is the number of retrieved documents for a given query and *α* is the parameter used to weight the importance of the retrieved documents.

### 2.7. Measures

In order to evaluate results, trec_eval (http://trec.nist.gov/) is used. It makes it possible to obtain several measures related to information retrieval [30]. The most commonly used are the following.

#### 2.7.1. Average Precision

For systems that return a ranked sequence of documents, it is preferable to consider the order in which the returned documents are presented. This measure averages the precision values from the rank positions where a relevant document is retrieved:

(9) $$\begin{array}{c} \text{Ave}\;P=\frac{\sum_{r=1}^{N}\left(P\left(r\right)\times \mathrm{rel}\left(r\right)\right)}{C_{r}}; \end{array}$$

- *r* is the rank;
- *N* is the number of documents retrieved;
- *rel*⁡(*r*) is a binary function on the relevance of a given rank;
- *P*(*r*) is the precision (proportion of a retrieved set that is relevant) at a given cut-off rank.

#### 2.7.2. Mean Average Precision (MAP)

It summarizes rankings from multiple queries by averaging average precision:

(10) $$\begin{array}{c} \text{MAP}=\frac{\sum_{q=1}^{Q}\text{Ave}P\left(q\right)}{\left|Q\right|}. \end{array}$$

#### 2.7.3. *R*-precision

*R*-precision (*R*-prec) is the precision after *R* documents have been retrieved, where *R* is the number of relevant documents for the topic.

## 3. Methods and Results

This section presents an overview of the tests performed, with respect to the processes of indexing, matching, and query expansion presented in Section 2.

### 3.1. Indexing Processes Testing

The first tests are based on a study of the benefits produced by the use of stemming and stopwords in the indexing of documents and queries.

We analyze the impact of stemming algorithms (Porter and Krovetz) and stopword lists (NLM and SMART) in the retrieval of documents from the corpus Cystic Fibrosis. The Okapi BM25 weighting algorithm is used with default parameters (*k*_1_ = 1.2, *b* = 0.75, and *k*_3_ = 7) and is applied to the Abstract field.

In Table 4 we can see that stemming is an effective technique to improve MAP. The performances are usually different between weak (Krovetz) and strong (Porter) stemming methods [12, 31, 32], but in our case the results are similar. In terms of MAP, strong stemming is a bit better, but in terms of *R*-prec, weak stemming is a bit better.

If we compare different stopword removal methods, we can see that removing stopwords improves the performance. From our experiments, using the largest stopword list (SMART) results is better than using the list with fewer stopwords. The Porter stemmer with SMART stopword list provides the best results. The last four combinations (highlighted with bold style) do not have significant differences, so we conduct the remaining tests with these four combinations.

### 3.2. Matching Processes Testing

#### 3.2.1. Parameterization of the Weighting Algorithms

The weighting algorithms used for ranking the retrieved documents in our tests are Okapi BM25 and TF-IDF, explained in Section 2.

In Okapi BM25, the values of the internal parameters *k*_1_, *k*_3_, and *b* should be adjusted based on the document collection and the type of queries where it is applied [2, 33–35]. A significant number of experiments have been done, and suggest general values of *k*_1_ and *k*_3_ between 1.2 and 2 (usually 1.2, although *k*_3_ is set between 7 and 1000 in the case of long queries) and *b* = 0.75 (although small values can sometimes report improvements).

Finding the set of optimal parameters is costly to compute, since they have local maxima that are singularity values [35]. Hence, we are using a simplistic optimization approach. The best values obtained in our tests with the Cystic Fibrosis corpora are *k*_1_ = 1.3, *b* = 0.6, and *k*_3_ = 1.2 (see Table 5).

For the TF-IDF weighting algorithm with Okapi TF formula, the parameters obtained for Okapi BM25 are the best approach, using the correspondence shown in Table 3. Moreover, the values obtained with the Log TF and Raw TF formulas without parameters were studied, verifying that they are worse than the approximation BM25 (see Table 6).

Many researchers use the BM25 algorithm in articles, steering their studies to retrieve information in several fields, not only in the Abstract [31, 36, 37]. By this assumption, we test how the MAP measure increases if we look for documents related to the queries in the Abstract, Title, and Mesh fields using the BM25, TF-IDF BM25, TF-IDF Log TF and TF-IDF Raw TF formulas (see Table 7).

The results obtained with Okapi BM25 are consistent with those presented by Trotman in [36, 37], which show a value of 0.2728 in the MAP measure with the same collection.

In Tables 6 and 7 we can see that the best MAP results are obtained using TF-IDF BM25 TF formula, so we continue our study with this approximation.

### 3.3. Query Expansion Processes Testing

#### 3.3.1. Pseudorelevance Feedback

To improve the results of the previous processes, we proceed to make pseudo relevance feedback using the Rocchio algorithm implemented in Lemur, explained in Section 2.

For the first time, it is necessary to parameterize the algorithm. The parameters for Rocchio are

 
*M* number of documents in feedback [10–100],
 
*K* number of terms selected in feedback [10–100], and
 
*α* coefficient adjustment (0,4].

Table 8 shows the best values of the parameters obtained for the Cystic Fibrosis collection.

After the parameterization, we test how the MAP increases if we look for documents related to the queries in the Abstract, Title, and Mesh fields, using the TF-IDF BM25 weighting algorithm (see Table 9). These results are comparable with those obtained by Shin and Han in their expansion system presented in [8], which achieved a maximum value of 0.35 for *R*-prec, with our *R*-prec being greater than 0.37.

#### 3.3.2. Use of MeSH to Expand Queries

As many authors have already worked with MeSH fields to retrieve information, we focus this part of the research on testing its efficiency in query expansion. Our method is based on the work of Kwangcheol Shin and Han [8], which proves the advantage of using MeSH Headings to expand the queries instead of working with terms not related to the MeSH fields.

The new strategy consists of expanding the queries with the MeSH Headings related to its terms. For the first time, we analyze the query and extract its keywords. Each keyword is automatically introduced at the PubMed online tool to match it with the Entry terms stored in MEDLINE. If the keyword is an Entry Term, we search the associated MeSH Headings (major and minor). Finally, the query is reformulated adding the descriptors contained in the MeSH fields extracted in the previous step.

In Table 10 the results of the experiment with the proposed strategy are shown. The query expansion is applied in the Abstract, Title, and MeSH fields of the documents and the weighting algorithm is TF-IDF BM25. As shown, the results are similar to those obtained with pseudo relevance feedback (Table 9), and are comparable with those obtained by Shin and Han in their expansion system [8].

**Figure 2:**
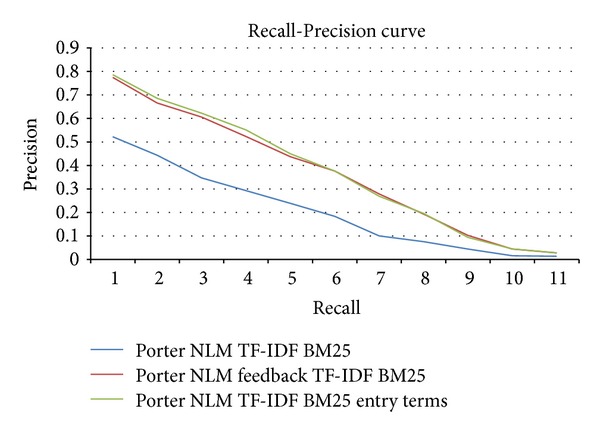
Recall-Precision curve obtained with the query expansion methods using the MeSH, Abstract, and Title fields.

Finally, a Recall-Precision graph (see Figure 2) is included showing the improvement obtained with the query expansion methods using the MeSH, Abstract, and Title fields (Tables 9 and 10) with respect to the document retrieval using only the Abstract field with TF-IDF BM25 (see Table 7). The figure shows that curves of the query expansion algorithms for the combination Porter stemmer-NLM stopwords are closest to the upper right hand corner of the graph (where recall and precision are maximized), which indicates the best performance.

## 4. Conclusions

We have developed and evaluated preprocessing and query expansion techniques for retrieving documents in several fields of biomedical articles belonging to the corpus Cystic Fibrosis, a corpus of MEDLINE documents. We test the benefit of using stemming and stopwords in the preprocessing of documents and queries, following the investigations of other authors.

Studies were carried out to compare the weighting algorithms Okapi BM25 and TF-IDF available in the Lemur tool, concluding that TF-IDF with tf formula given by BM25 approximation is superior in its results.

Document retrieval based on Abstract, MeSH, and Title fields seems more effective than looking at each of these fields individually. In addition, the use of relevance feedback, a technique widely used by researchers in this field, produces a great improvement in the retrieval of scientific documents. The Rocchio algorithm allows obtaining good results, improving MAP and other measures.

Finally, we perform a study to improve searching expanding queries with MeSH terms. For this, we have enhanced queries locating Entry terms in them and obtaining MeSH Headings in PubMed in order to expand the original query and to map it with the documents. The results are good, making use of the Title, Abstract and MeSH fields to improve the list of documents retrieved, compared to baseline methods.

In this paper, authors have used a very simplistic approach to determine the BM25 parameters values. Tuning the BM25 free parameters (*k*_1_, *b*, and *k*_3_) is a difficult and computationally expensive problem that requires advanced multidimensional optimization techniques. Retrieval accuracy can be improved using more advanced parameterization methods.

## Figures and Tables

**Table 1:** A sample of a MEDLINE document.

TI	The occurrence of Cystic Fibrosis and celiac sprue within a single sibship.
MJ	CYSTIC-FIBROSIS: fg. CELIAC-DISEASE: fg.
MN	ADULT. BIOPSY. CELIAC-DISEASE: co, fg. CHILD. CYSTIC-FIBROSIS: co.
	DIET-THERAPY. DILATATION. FEMALE. FLOCCULATION. GLUTEN: me.
	HUMAN. INTESTINAL-MUCOSA: pa, ra. INTESTINE-SMALL: pa, ra.
	JEJUNUM: pa. MALE. PEDIGREE. CELIAC-DISEASE: co, th.
AB	A family is presented in which celiac sprue and cystic fibrosis occurred within the
	same sibship. A cousin of the index case was also discovered to have celiac sprue.
	The genetics and incidence of both conditions are reviewed. It is estimated that
	the likelihood of this association occurring on the basis of chance in this is 1 in 50,000.

**Table 2:** A sample of query with its relevant documents and relevance scores.

QU	What is the association between liver disease (cirrhosis) and vitamin A metabolism in CF?

RD	165 1122 174 0001 362 0001 370 0001 414 2222 443 0100 794 2110 992 1010 1040 0001 1115 0102

**Table 3:** Correspondence between parameters of the BM25 weighting and Okapi TF.

	Okapi BM25	Okapi TF
tf_*d*_	*k*_1_	*k*_1_
tf_*d*_ and tf_*q*_	*b*	*b*
tf_*q*_	*k*_3_	*k*_1_

**Table 4:** Accuracy of query search using different stemming functions and stopword lists in Cystic Fibrosis. Evaluation measures used are MAP (mean average precision), *R*-prec (*R* precision), and *D*_*r*_ (number of relevant documents retrieved).

Combinations	Measures		
	MAP	*R*-prec	*D*_*r*_
Baseline	0.1545	0.2098	683
Porter stemmer	0.1663	0.2154	747
Krovetz stemmer	0.1663	0.2231	740
NLM stopword list	0.1681	0.2242	723
SMART stopword list	0.1695	0.2243	728
Porter stemmer-NLM stopwords	**0.1790**	**0.2332**	**786**
Porter stemmer-SMART stopwords	**0.1808**	**0.2342**	**790**
Krovetz stemmer-NLM stopwords	**0.1799**	**0.2333**	**782**
Krovetz stemmer-SMART stopwords	**0.1808**	**0.2324**	**786**

The bold font refers to the best values for the parameters.

**Table:** (a) The best value obtained for the *k*_1_ parameter

Combinations	Parameters																				
	*k*_1_	*b*	*k*_3_	*k*_1_	*b*	*k*_3_	*k*_1_	*b*	*k*_3_	*k*_1_	*b*	*k*_3_	*k*_1_	*b*	*k*_3_	*k*_1_	*b*	*k*_3_	*k*_1_	*b*	*k*_3_
	0	0.75	1.2	1	0.75	1.2	2	0.75	1.2	1.5	0.75	1.2	**1.3**	0.75	1.2	1.2	0.75	1.2	1.4	0.75	1.2
Porter stemmer-NLM stopwords	0.1521			0.1803			0.1792			0.1792			**0.1807**			0.1798			0.1792		
Porter stemmer-SMART stopwords	0.1531			0.1820			0.1783			0.1788			**0.1813**			0.1815			0.1807		
Krovetz stemmer-NLM stopwords	0.1521			0.1797			0.1778			0.1802			**0.1804**			0.1806			0.1799		
Krovetz stemmer-SMART stopwords	0.1557			0.1828			0.1793			0.1801			**0.1824**			0.1815			0.1819		

The bold font refers to the best values for the parameters.

**Table:** (b) The best value obtained for the *b* parameter

Combinations	Parameters																				
	*k*_1_	*b*	*k*_3_	*k*_1_	*b*	*k*_3_	*k*_1_	*b*	*k*_3_	*k*_1_	*b*	*k*_3_	*k*_1_	*b*	*k*_3_	*k*_1_	*b*	*k*_3_	*k*_1_	*b*	*k*_3_
	1.3	0	1.2	1.3	1	1.2	1.3	0.75	1.2	1.3	0.65	1.2	1.3	**0.6**	1.2	1.3	0.55	1.2	1.3	0.70	1.2
Porter stemmer-NLM stopwords	0.1652			0.1715			0.1807			0.1810			**0.1824**			0.1821			0.1799		
Porter stemmer-SMART stopwords	0.1669			0.1695			0.1813			0.1816			**0.1814**			0.1808			0.1817		
Krovetz stemmer-NLM stopwords	0.1667			0.1706			0.1804			0.1802			**0.1819**			0.1819			0.1795		
Krovetz stemmer-SMART stopwords	0.1701			0.1707			0.1824			0.1823			**0.1824**			0.1821			0.1818		

The bold font refers to the best values for the parameters.

**Table:** (c) The best value obtained for the *k*_3_ parameter.

Combinations	Parameters																				
	*k*_1_	*b*	*k*_3_	*k*_1_	*b*	*k*_3_	*k*_1_	*b*	*k*_3_	*k*_1_	*b*	*k*_3_	*k*_1_	*b*	*k*_3_	*k*_1_	*b*	*k*_3_	*k*_1_	*b*	*k*_3_
	1.3	0.6	0	1.3	0.6	1	1.3	0.6	2	1.3	0.6	7	1.3	0.6	1.5	1.3	0.6	**1.2**	1.3	0.6	1.3
Porter stemmer-NLM stopwords	0.1824			0.1825			0.1824			0.1817			0.1823			**0.1824**			0.1822		
Porter stemmer-SMART stopwords	0.1814			0.1813			0.1813			0.1810			0.1814			**0.1814**			0.1814		
Krovetz stemmer-NLM stopwords	0.1811			0.1819			0.1825			0.1815			0.1822			**0.1819**			0.1819		
Krovetz stemmer-SMART stopwords	0.1817			0.1822			0.1820			0.1815			0.1824			**0.1824**			0.1824		

The bold font refers to the best values for the parameters.

**Table 6:** MAP values for the TF-IDF BM25, Raw TF, and log⁡TF formulas.

Combinations	Parameters										
	*k*_1_	*b*	*k*_3_	*k*_1_	*b*	*k*_3_	*k*_1_	*b*	*k*_3_	Raw TF	log⁡TF
	1.2	0.75	1000	1.3	0.6	1.2	1.2	0.7	1.2		
Porter stemmer-NLM stopwords	0.1861			**0.1868**			0.1861			0.1422	0.1749
Porter stemmer-SMART stopwords	0.1866			**0.1898**			0.1878			0.1445	0.1742
Krovetz stemmer-NLM stopwords	0.1821			**0.1843**			0.1827			0.1420	0.1736
Krovetz stemmer-SMART stopwords	0.1828			**0.1866**			0.1839			0.1436	0.1731

The bold font refers to the best values for the parameters.

**Table 7:** MAP values to retrieving in Abstract, Title, and MeSH fields using different weighting algorithms.

Combinations	Algorithms			
	BM25	TF-IDF BM25	log⁡TF	Raw TF
Porter stemmer-NLM stopwords	0.2717	**0.2953**	0.2683	0.2209
Porter stemmer-SMART stopwords	0.2733	**0.2930**	0.2665	0.2221
Krovetz stemmer-NLM stopwords	0.2719	**0.2929**	0.2684	0.2208
Krovetz stemmer-SMART stopwords	0.2737	**0.2904**	0.2654	0.2228

The bold font refers to the best values for the parameters.

**Table:** (a) The best value for the *M* parameter

Combinations	Parameters												
	*M*	*K*	*α*	*M*	*K*	*α*	*M*	*K*	*α*	*M*	*K*	*α*	Baseline
	**10 **	10	0.5	5	10	0.5	15	10	0.5	30	10	0.5	
Porter stemmer-NLM stopwords	**0.2079**			0.1991			0.2070			0.2053			0.1868
Porter stemmer-SMART stopwords	**0.2075**			0.2026			0.2053			0.2052			0.1898
Krovetz stemmer-NLM stopwords	**0.2094**			0.1974			0.2007			0.2025			0.1843
Krovetz stemmer-SMART stopwords	**0.2074**			0.1998			0.2022			0.2056			0.1866

The bold font refers to the best values for the parameters.

**Table:** (b) The best value for the *K* parameter

Combinations	Parameters														
	*M*	*K*	*α*	*M*	*K*	*α*	*M*	*K*	*α*	*M*	*K*	*α*	*M*	*K*	*α*
	10	10	0.5	10	20	0.5	10	30	0.5	10	40	0.5	10	**28**	0.5
Porter stemmer-NLM stopwords	0.2079			0.2103			0.2117			0.2102			**0.2116**		
Porter stemmer-SMART stopwords	0.2075			0.2083			0.2105			0.2087			**0.2100**		
Krovetz stemmer-NLM stopwords	0.2094			0.2068			0.2075			0.2068			**0.2093**		
Krovetz stemmer-SMART stopwords	0.2074			0.2061			0.2015			0.2032			**0.2033**		

The bold font refers to the best values for the parameters.

**Table:** (c) The best value for the *α* parameter

Combinations	Parameters														
	*M*	*K*	*α*	*M*	*K*	*α*	*M*	*K*	*α*	*M*	*K*	*α*	*M*	*K*	*α*
	10	28	**0.5**	10	28	0.1	10	28	1	10	28	0.9	10	28	0.4
Porter stemmer-NLM stopwords	**0.2116**			0.1957			0.2103			0.2099			0.2105		
Porter stemmer-SMART stopwords	**0.2100**			0.1956			0.2035			0.2091			0.2093		
Krovetz stemmer-NLM stopwords	**0.2093**			0.1904			0.2072			0.2097			0.2089		
Krovetz stemmer-SMART stopwords	**0.2033**			0.1932			0.2024			0.2031			0.2061		

The bold font refers to the best values for the parameters.

**Table 9:** Evaluation measures using the pseudorelevance feedback in Abstract, Title, and MeSH fields.

Combinations	Measures	
	MAP	*R*-Prec
Porter stemmer-NLM stopwords	0.3468	0.3780
Porter stemmer-SMART stopwords	0.3391	0.3731
Krovetz stemmer-NLM stopwords	0.3475	0.3834
Krovetz stemmer-SMART stopwords	0.3435	0.3790

**Table 10:** Evaluation measures using query expansion with descriptors, applied in MeSH, Title, and Abstract fields.

Combinations	Measures	
	MAP	*R*-prec
Porter stemmer-NLM stopwords	0.3538	0.3791
Porter stemmer-SMART stopwords	0.3489	0.3766
Krovetz stemmer-NLM stopwords	0.3465	0.3750
Krovetz stemmer-SMART stopwords	0.3424	0.3732
